# Association of Promoter Methylation of *RUNX3* Gene with the Development of Esophageal Cancer: A Meta Analysis

**DOI:** 10.1371/journal.pone.0107598

**Published:** 2014-09-17

**Authors:** Yi Wang, Xiuguang Qin, Jieqing Wu, Bo Qi, Yipeng Tao, Wenju Wang, Fulei Liu, Hanchen Li, Baosheng Zhao

**Affiliations:** 1 Department of Thoracic Surgery, The First Affiliated Hospital of Xinxiang Medical University, Weihui, Henan, P. R. China; 2 Department of Oncology, The First Affiliated Hospital of Xinxiang Medical University, Weihui, Henan, P. R. China; 3 Department of Gastroscopy, The First Affiliated Hospital of Xinxiang Medical University, Weihui, Henan, P. R. China; Duke Cancer Institute, United States of America

## Abstract

**Background:**

Runt-related transcription factor 3 (*RUNX3*) is a member of the runt-domain family of transcription factors. Emerging evidence indicates that RUNX3 is a tumor suppressor gene in several types of human cancers including esophageal cancer. However, the association between *RUNX3* promoter methylation and esophageal cancer remains unclear. Here we conducted a systematic review and meta-analysis to quantitatively evaluate the effects of *RUNX3* promoter methylation on the incidence of esophageal cancer.

**Methods:**

A detailed literature search was made on Medline, Pubmed and Web of Science for related research publications written in English and/or Chinese. Methodological quality of the studies was also evaluated. The data were extracted and assessed by two reviewers independently. Analysis of pooled data were performed, the odds ratios (OR) were calculated and summarized respectively.

**Results:**

Final analysis of 558 patients from 9 eligible studies was performed. The result showed that *RUNX3* methylation was significantly higher in esophageal cancer than in normal squamous mucosa from the proximal resection margin or esophageal benign lesions (OR = 2.85, CI = 2.01–4.05, P<0.00001). The prevalence of lymph node involvement, tumor size (T1–T2 vs T3–T4) and histological grade was significantly greater in *RUNX3*-negative cases (*RUNX3* unmethylated groups) than in *RUNX3*-positive cases (OR = 0.25, CI = 0.14–0.43, P<0.00001). *RUNX3* methylation was significantly higher in esophageal adenocarcinoma (EAC) than Barrett’s esophagus (OR = 0.35, CI = 0.20–0.59, P<0.0001). In addition, the pooled HR for overall survival (OS) showed that decreased *RUNX3* expression was associated with worse survival in esophageal cancer (HR = 4.31, 95% CI = 2.57–7.37, P<0.00001).

**Conclusions:**

The results of this meta-analysis suggest that *RUNX3* methylation is associated with an increased risk, progression as well as worse survival in esophageal cancer. *RUNX3* methylation, which induces the inactivation of *RUNX3* gene, plays an important role in esophageal carcinogenesis.

## Introduction

Esophageal cancer is the eighth most common cancer worldwide, esophageal squamous cell carcinoma (ESCC) and adenocarcinoma (EAC) are two major histopathological types of esophageal cancer [1], [2]. It has been reported that esophageal tumor currently affects more than 450, 000 people worldwide and the incidence is still increasing [1]. Surgery is the standard therapy for esophageal tumor [3]. However, the overall prognosis is far less satisfactory and pretty modest, with 5-year survival rates ranging between 15 and 50% [4], [5]. Therefore, investigating the mechanism of initiation and progression and finding out the therapeutic targets at biomolecular levels are highly desired for the treatment of esophageal cancer.

The Runt-related transcription factor 3 (*RUNX3*) gene is a tumor suppressor gene involved in the TGF-β signaling pathway, which was cloned and identified as a human runt-domain containing gene in 1994 [6]. Its precise function has been intensively studied in gastric cancer, with upregulation inducing cell cycle arrest, apoptosis, and down regulating cyclin D1 expression [7], [8], [9], [10], however, its role in esophageal cancer has not been thoroughly investigated and reviewed. Inactivation of *RUNX3* by promoter methylation has been found to play an important role during normal tissue development and in tumorigenesis in esophagus [11]. In this study, we reviewed and performed a meta analysis on the published clinical studies regarding the effect of *RUNX3* on patients with esophageal cancer.

## Materials and Methods

### Search strategy

Medline, Pubmed and Web of Science were searched in December 2013 using the search terms: “esophageal” and “cancer or tumor or neoplasm or carcinoma” and “RUNX3”. Studies identified through the approaches as described above were screened by titles first, then abstracts of the publications. After exclusion of non-relevant publications and identifications of duplicates from the different databases, the remaining papers were evaluated in the full text version for in- and exclusion criteria and for relevant articles in the reference lists. All clinical studies except case reports were chosen. The language of publication was restricted to English and Chinese. All searched data were retrieved. Authors’ bibliographies and references of selected studies were also searched for other relevant studies. The most complete study was chosen to avoid duplication if same patient populations were reported in several publications.

### Selection criteria

We collected all eligible articles about relationship between *RUNX3* methylation and/or expression and clinicopathological features and clinical outcomes in esophageal cancer in this meta-analysis. Studies meeting the following inclusion criteria were included: (1) *RUNX3* methylation and/or expression evaluated in the circulation and/or primary esophageal cancer tissues, (2) studies revealing the relationship between *RUNX3* methylation and/or expression and esophageal cancer clinicopathological parameters and prognosis, (3) *RUNX3* methylation and/or expression examined by polymerase chain reaction (PCR), (4) articles published as a full paper in English, (5) studies providing sufficient information to estimate hazard ratio (HR) about overall survival (OS) and 95% confidence interval (CI). The exclusion criteria included the following: (1) letters, reviews, case reports, conference abstracts, editorials, expert opinion, non-English, non-Chinese language papers; (2) articles with no information on OS or insufficient information for calculation of HR; and (3) all publications regarding *in vitro*/*ex vivo* studies, cell lines and human xenografts.

#### Data extraction

Two investigators independently extracted data from eligible studies. Disagreements were resolved by discussion and consensus. Two investigators reviewed all of articles that fit inclusion and exclusion criteria. The following information was recorded for each study: the first author name, year of publication, sample source, number of cases, clinicopathological parameters, cancer with tumor node metastasis (TNM) stage, *RUNX3* methylation and/or expression, and patient survival. Data for study characteristics and clinical response were summarized and turned the data into table format. Heterogeneity of investigation was evaluated to determine whether the data of the various studies were appropriate for a meta-analysis.

#### Statistical analysis

Analysis was conducted using the Stata 12.0 (Stata Corporation, TX, USA) and Review Manager 5.2 (Cochrane Collaboration, Oxford, UK). Comparisons of dichotomous measures were done by pooled estimates of odds ratios (ORs) as well as their 95% CIs. *P* value of <0.05 was considered to be statistically significant. Heterogeneity was examined by a chi-square test with significance set at *P*<0.10; the total variation among studies was estimated by I square. If there was heterogeneity among studies, we used a random effect model to pool the ORs; otherwise, a fixed effect model was chosen.

**Figure 1:**
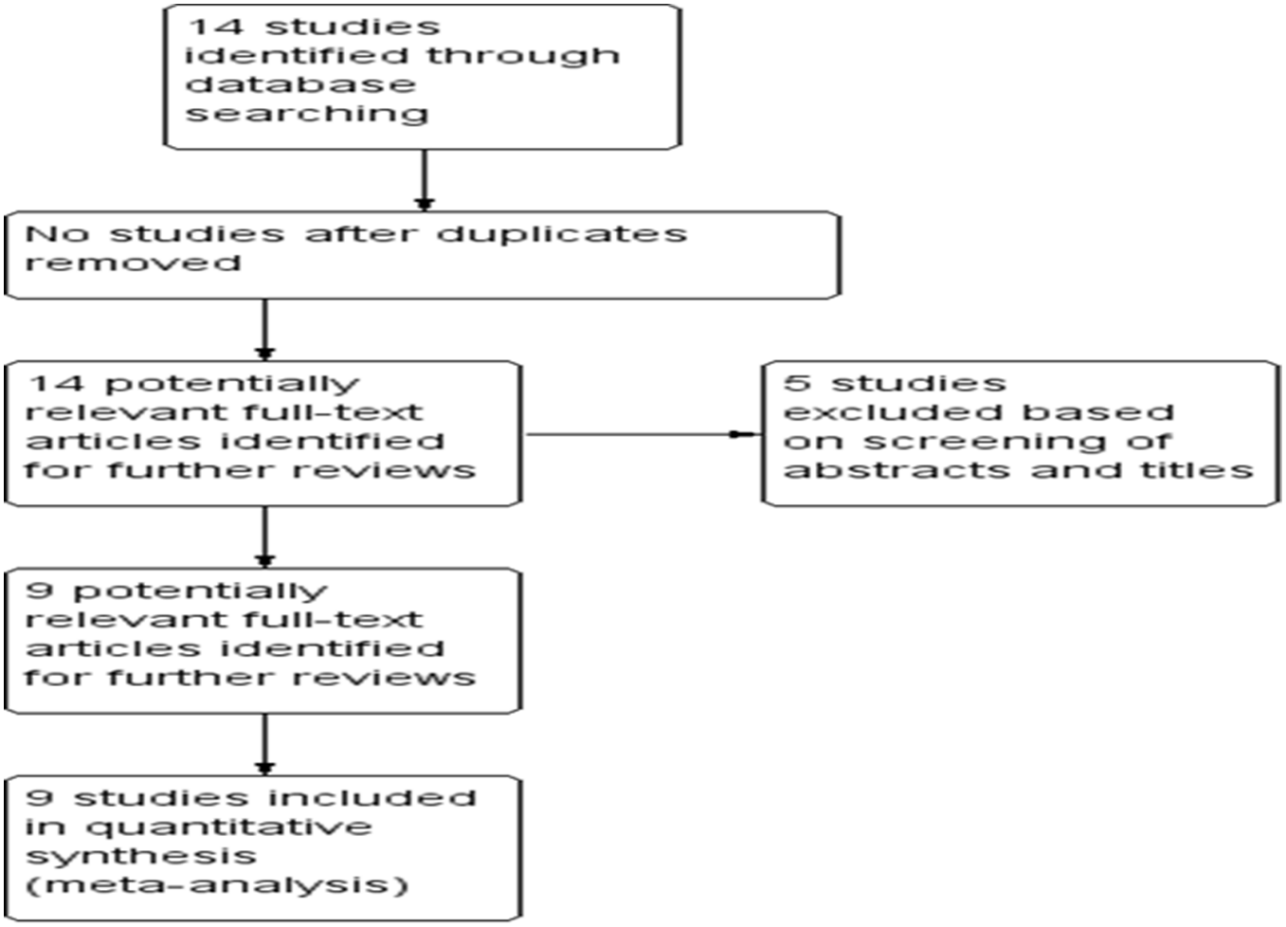
Flow chart of study selection.

The database search generated 14 articles from Pubmed and the Web of Science. After initial screening of all titles, abstracts and eligibility, 9 full-text studies were retracted for more detailed assessment. The search of the article references did not produce additional publications. Eventually, 9 publications met the inclusion criteria for qualitative study and for meta-analysis. The article search and study selection is depicted in Figure 1.

## Results

### Identification of relevant studies

Fourteen publications were identified by using the search method as described above. Five of those were excluded due to laboratory studies, non-original articles (review), or studies irrelevant to the current analysis. Eventually, there were nine studies included in the final meta-analysis [11], [12], [13], [14], [15], [16], [17], [18], [19] (Figure. 1).

### Study characteristics

Nine studies published from 2005 to 2013 were eligible for meta-analysis. A total of 558 patients including esophageal squamous cell carcinomas (ESCCs) and esophageal adenocarcinomas (EACs) from China, Japan, Australia and USA were enrolled. A total of 140 cases of Barrett’s esophagus (BE) were also included in this analysis. Their basic characteristics are summarized in Table 1.

**Table 1:** Basic characteristics of the included studies.

Study/Country	Patients	Methods	Primary Aim	Methylationsite	RUNX3 expression
Hiramatsu et al2005 [17]	69	RT-PCR,Western blot	Eexamine the expression of RUNX3protein in human esophageal mucosaand squamous cell carcinoma incomparison with clinicopathological profiles		+
Schulmann et al2005 [13]	77	Methylationspecic PCR (MSP)	Determine at what stage,inactivation of *RUNX3* occursand predicts prognosis	Promoter,CpG islands	+
Long et al2007 [19]	42	RT-PCR,MSP	Determine whether promotermethylation of the *RUNX3*gene correlates with ESCCtumor progression	Promoter,CpG islands	+
Sakakura et al2007 [34]	51	MSP	Determine whether *RUNX3*expression is associated withradioresistance and prognosis	Promoter,CpG islands	+
Tonomoto et al2007 [18]	61	qRT-PCR,MSP	Detemine whetherthe precise expression, prognosticimpact and methylation status ofRUNXs in esophageal squamouscell carcinoma.	Promoter,CpG islands	+
Smith et al2008 [15]	37	MSP	Determine at what stage,in the progression fromBE to EAC, methylation ofkey genes occurs.	Promoter,CpG islands	+
Sugiura et al2008 [12]	70	qRT-PCR,MSP	Determine whether *RUNX3*expression is correlated withESCC progression and prognosis	Promoter,CpG islands	+
Liu et al2011 [11]	81	MSP	Determine the effects of plasmaDNA methylation of *RUNX3*on recurrence of ESCC	Promoter,CpG islands	+
Zheng et al2011 [16]	70	MSP	Evaluate the diagnostic roleof *RUNX3* gene methylation inserum DNA of esophageal squamouscell carcinoma (ESCC), gastriccancer (GC) and colorectalcancer (CRC) patients.	Promoter,CpG islands	+

### RUNX3 methylation and expression and clinicopathological features

#### 1. Inactivation of *RUNX3* through methylation in esophageal cancers

**Figure 2:**
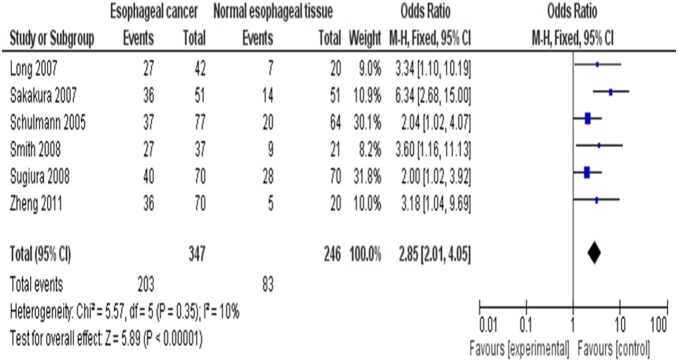
The pooled OR from 6 studies including 347 esophageal cancers and 246 normal squamous mucosa OR = 2.85, CI = 2.01–4.05, P<0.00001.

It was reported that the loss of *RUNX3* mRNA expression was statistically correlated with the promoter hypermethylation in esophageal tumors (*P*<0.001) [18], [19]. We observed that *RUNX3* methylation was significantly higher in ESCC/EAC than in normal squamous mucosa from the proximal resection margin or esophageal benign lesions. The pooled OR from 6 studies including 347 esophageal cancers and 246 normal squamous mucosa was shown in Figure 2 (OR = 2.85, CI = 2.01–4.05, P<0.00001), which indicated that *RUNX3* inactivation through methylation plays an important role in the pathogenesis of esophageal cancers.

#### 2. Role of *RUNX3* methylation in esophageal cancer development

**Figure 3:**
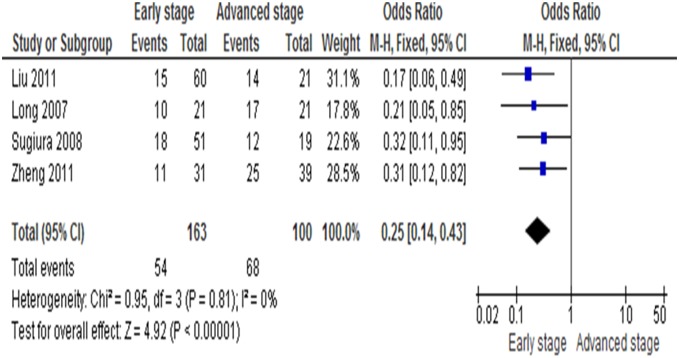
Forest plot of odds ratio (OR) in 263 patients pooled in 4 studies in serum/cancer tissues DNA from advanced stage, including tumor size (T1–T2 vs T3–T4), lymph node involvement, lymph and blood vessels metastasis, and recurrence in esophageal carcinomas. The prevalence of lymph node involvement, tumor size (T1–T2 vs T3–T4) and histological grade was significantly greater in *RUNX3*-negative cases (*RUNX3* unmethylated group) than in *RUNX3*-positive cases, OR = 0.25, CI = 0.14–0.43, P<0.00001.

We analyzed 263 patients pooled in 4 studies to assess whether the aberrant *RUNX3* methylation/expression in serum/cancer tissues DNA was associated with advanced stage, including tumor size (T1–T2 vs T3–T4), lymph node involvement, lymph and blood vessels metastasis, and recurrence in esophageal carcinomas. *RUNX3* methylation/expression estimated in biopsy/blood samples and clinicopathological factors as described above were examined. The prevalence of lymph node involvement, tumor size (T1–T2 vs T3–T4) and histological grade was significantly greater in *RUNX3*-negative cases (*RUNX3* unmethylated groups) than in *RUNX3*-positive cases (Figure 3), with OR = 0.25, CI = 0.14–0.43, *P*<0.00001. These results suggest that epigenetic silencing of *RUNX3* gene expression by promoter hypermethylation may play an important role in esophageal cancer progression and development. With similarity in pathologic stage, Hiramatsu et al [17] showed that *RUNX3* expression was significantly higher in 19 well-differentiated ESCCs than in 56 moderately or 69 poorly differentiated ESCCs (p<0.01).

**Figure 4:**
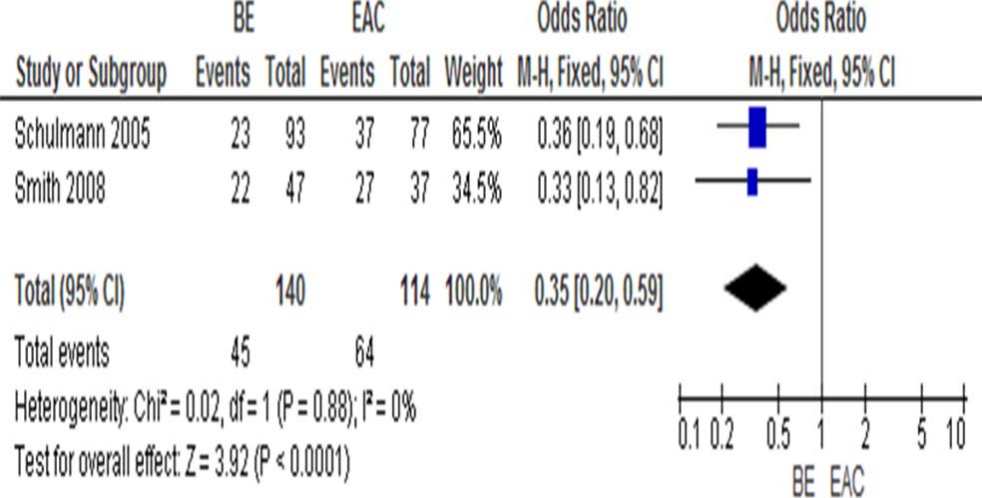
*RUNX3* methylation significantly higher in esophageal adenocarcinoma (EAC) than Barrett’s esophagus (BE), OR = 0.35, CI = 0.20–0.59, P<0.0001.

Barrett's esophagus (BE) is the metaplastic replacement of squamous with columnar epithelium in the esophagus, as a result of reflux. It is a major risk factor for the development of EAC [20], [21]. We observed that *RUNX3* methylation was significantly higher in EAC than in BE as shown in Figure 4, OR = 0.35, CI = 0.20–0.59, *P*<0.0001. *RUNX3* hypermethylation is an independent risk factor for progression of BE to high-grade dysplasia of esophagus and EAC [13], [22].

No heterogeneity was observed in the analysis of *RUNX3* methylation/low expression in normal samples and esophageal patient samples (*P* = 0.81), in BE and EAC patients (*P* = 0.88). There is no heterogeneity of *RUNX3* methylation with advanced stage (*P* = 0.81), so the fixed effect model was used.

#### 3. *RUNX3* as a prognostic factor for esophageal cancer

**Figure 5:**
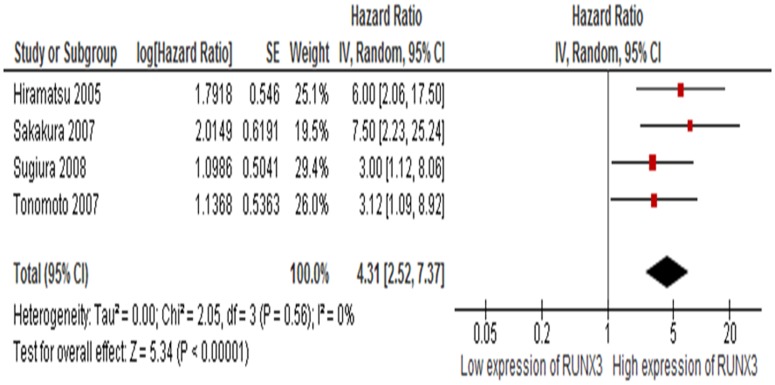
All four included studies estimated the relationship between OS and *RUNX3* methylation/expression. The pooled HR for OS showed that decreased *RUNX3* expression was associated with worse survival in esophageal cancer, HR = 4.31, 95% CI = 2.57–7.37, P<0.00001.

Four studies included investigated the relationship between OS and *RUNX3* methylation/expression. The pooled HR for OS showed that decreased *RUNX3* expression was associated with worse survival in esophageal cancer as shown in Figure 5 (HR = 4.31, 95% CI = 2.57–7.37, *P*<0.00001).

#### 4. Sensitivity analyses and publication bias

**Figure 6:**
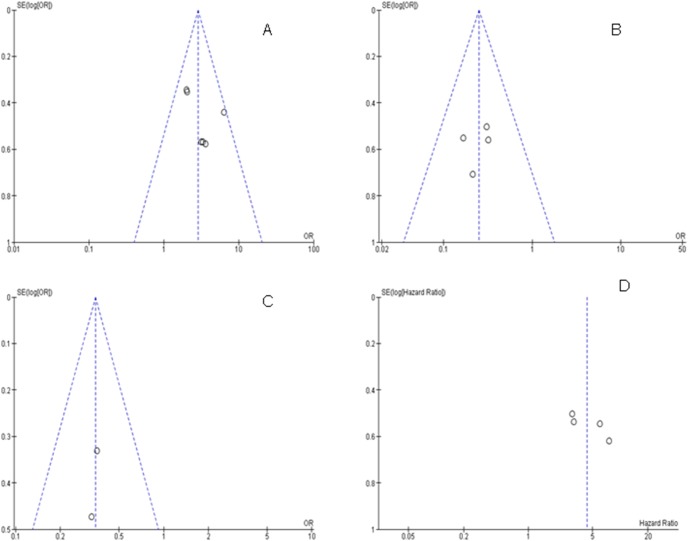
The funnel plots were largely symmetric suggesting there were no publication biases in the meta-analysis of *RUNX3* methylation/expression and clinicopathological features as well as overall survival respectively. The funnel plot from 6 studies comparing esophageal cancers and normal squamous mucosa (A). The funnel plot from 4 studies in determining *RUNX3* hypermethylation in advanced stage (T3–T4) and early stage (T1–T2) (B). The funnel plot from 2 studies in determining *RUNX3* hypermethylation in Barrett’s esophagus (BE) and esophageal adenocarcinoma (EAC) (C). The funnel plot from 4 studies in determining the relationship between *RUNX3* hypermethylation and overall survival (OS) in esophageal cancer (D).

A sensitivity analysis, in which one study was removed at a time, was conducted to assess the result stability. The pooled ORs and HRs were not significantly changed, indicating the stability of our analyses. The funnel plots were largely symmetric (Figure 6) suggesting there were no publication biases in the meta-analysis of *RUNX3* methylation/expression and clinicopathological features as well as overall survival respectively.

## Discussion

*RUNX3* belongs to the runt domain family of transcriptional factors that plays an important role during normal tissue development and in tumorigenesis [7], [23], [24], [25], [26], [27], [28], [29], [30], [31], [32], [33]. *RUNX3* inactivation is a crucial factor to determine cancer pathogenesis and clinical outcome in a variety of cancer types [23]. The different modes of *RUNX3* inactivation in various tumor types include hemizygous deletion, mutations, hypermethylation, histone modifications and cytoplasmic mislocalization [23]. To date, there have been some studies describing the precise expression, prognostic impact and methylation status of *RUNX3* in esophageal cell carcinoma [13], [16], [17], [19], [33], [34]. We conducted a meta-analysis to evaluate the correlation between *RUNX3* promoter methylation/low expression and esophageal cancer. Analysis of the pooled data showed that 1) Esophageal cancers had a higher methylation rate than normal tissues, or well-differentiated cancer tissues; 2) The prevalence of lymph node involvement, tumor size (T1–T2 vs T3–T4) and histological grade was significantly greater in *RUNX3*-negative cases (*RUNX3* unmethylated groups) than in *RUNX3*-positive cases; *RUNX3* methylation was significantly higher in EAC than in BE; and 3) The pooled HR for OS showed that decreased *RUNX3* expression was associated with worse survival in esophageal cancer.

ESCC and EAC are the two major histological types of esophageal carcinoma. ESCC occurs mostly in the endemic areas such as Asia and Africa, whereas EAC is the most common in North America and Europe [35]. Although these two esophageal cancers are different in pathogenesis, epidemiology, tumor biology, prognosis and therapeutic strategies among the pooled patients in this meta analysis, *RUNX3* methylation/low expression was associated with the pathogenesis of both types of esophageal cancer, indicating that *RUNX3* might be responsible for different patterns of tumorigenesis.

Patients with BE have an increased risk of developing EAC. The most established marker for the risk of developing EAC in BE is dysplasia [36]. Because of not well defined natural history of low grade dysplasia (LGD), not well characterized histological classification of dysplasia, moreover especially extremely high interobserver variability for LGD, molecular biomarkers are needed to improve the risk classification of BE patients [37], [38], [39]. It was accepted that BE is a precancerous tissue, and that aberrant promoter methylation occurs early in metaplasia before histological evidence of progression to cancer. Some underlying mechanisms for aberrant DNA methylation in Barrett’s metaplasia have been noted. Frequent *RUNX3* inactivation through promoter hypermethylation was reported in ESCC, EAC, Barrett’s metaplasia and dysplasia [13], [15], [17], [18]. The result of this meta analysis indicated that the methylation of *RUNX3* gene was significantly higher in both BE and EAC than in the squamous samples, that metaplasia BE was nearly as abnormal epigenetically as EAC. In other words, the aberrant methylation of *RUNX3* gene is an early event, which most probably occurs independently of EAC [13], [15].

*RUNX3* exerts pleiotropic effects during tumor suppression. It inhibits the oncogenic Wnt signaling pathway via formation of a complex with the TCF4-β-catenin complex and hampering it from binding to target genes such as c-myc and cyclin D1 [23], [40]. RUNX3 interacts with SMAD3/SMAD4 to regulate TGF-β-dependent inhibition of proliferation and apoptosis by activation of p21 and Bim. Sakakura et al [34] found that frequent silencing of *RUNX3* by promoter hypermethylation in esophageal squamous cell carcinomas is associated with radioresistance and poor prognosis. In other words, *RUNX3* gene expression promotes radiosensitivity, whereas its inactivation facilitates radioresistance [34]. They further confirmed that RUNX3 activates Bim expression and increases sensitivity to radiation and induces TGF-β-mediated apoptosis in ESCC cells, therefore functioning as a crucial determinant of radiosensitivity [34]. Thus, induction of RUNX3 expression by overcoming gene silencing may enhance radiosensitivity against tumor which may have crucial clinical impact for esophageal cancer patients. It is also exciting that measurement of RUNX3 expression status in pretreatment specimens may predict radiosensitivity [34].

Progression from BE to esophageal cancer appears to mirror the accumulation of genetic abnormalities, suggesting a stepwise progression of genetic changes in esophageal cancer. *RUNX3*, in combination of a panel of other genes that are inactivated by methylation, can be developed as biomarkers for various tissues. This is becoming a good strategy for risk stratification to predict neoplastic progression including esophageal cancer [14].

Consistent results were shown in sensitivity analyses, and no evidence of publication bias was found. However, this study has several potential limitations. First, the possibility of information and selection biases and unidentified confounders could not be completely excluded because all of the included studies were observational. Second, the searching strategy was restricted to articles published in English and Chinese. Articles with potentially high-quality data that were published in other languages were not included because of anticipated difficulties in obtaining accurate medical translation. Hence, cautions should be taken when our findings are interpreted among the general populations.

In conclusion, our meta-analysis showed *RUNX3* may play an important role in esophageal cancer initiation and progression. Plasma levels of RUNX3 promoter hypermethylation may be a promising biomarker for the early diagnosis of esophagus squamous cell carcinoma [16]. In addition, *RUNX3* methylation is associated with an increased risk and worse survival in esophageal cancer patients. Further large-scale studies, especially multi-center and well-matched cohort research will provide more insight into the role of *RUNX3* in the prognosis and clinical implementation of esophageal cancer patients. The fact that silencing of *RUNX3* gene at the transcriptional level and functional inactivation at the protein level in esophagus cancer are strongly correlated with poor prognosis and may occur in early phase of tumor initiation, making it a promising target for therapeutic approaches. Maintaining *RUNX3* expression under microenvironment stress conditions, either directly or indirectly or reversing *RUNX3* silencing could be a new direction for drug discovery for esophageal cancers. Further, *RUNX3* was reported to control Notch signaling which is tightly linked to cancer stem cell (CSCs) [7]. Conventional chemotherapy can induce resistance to chemotherapeutic agents, and tumor regrowth mediated by CSCs, therefore targeting *RUNX3* gene and its related signaling pathways could be another mechanism for therapeutic approaches for cancer treatment aiming at CSC elimination.

## Supporting Information

Checklist S1 **PRISMA checklist.**
(DOC)

Flowchart S1 **PRISMA flowchart.**
(DOC)
