# Supplementary material for: Association of Promoter Methylation of RUNX3 Gene with the Development of Esophageal Cancer: A Meta Analysis
Source: PLoS One. 2014 Sep 17;9(9):e107598. doi: 10.1371/journal.pone.0107598 (PMC4167998; doi:10.1371/journal.pone.0107598)
Supplement: Flowchart S1 — PRISMA flowchart. (DOC) [file pone.0107598.s002.doc]

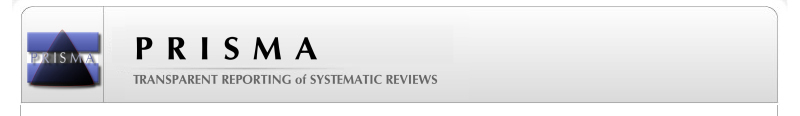
**PRISMA 2009 Flow Diagram**

**Screening**

**Included**

**Eligibility**

**Identification**

Records identified through database searching
(n = 14 )

Additional records identified through other sources
(n =0)

Records after duplicates removed
(n =14)

Records screened
(n = 14)

Records excluded
(n =0 )

Full-text articles assessed for eligibility
(n =14 )

Full-text articles excluded, with reasons
(n =5 )

Studies included in qualitative synthesis
(n = 9 )

Studies included in quantitative synthesis (meta-analysis)
(n = 9 )
